# Supplementary material for: Lesbian, gay, bisexual, transgender, intersex, and its legalisation in Africa: Insights from tertiary-level students in Ghana
Source: PLoS One. 2023 Jul 7;18(7):e0287726. doi: 10.1371/journal.pone.0287726 (PMC10328334; doi:10.1371/journal.pone.0287726)

REGRESSION OUTPUT


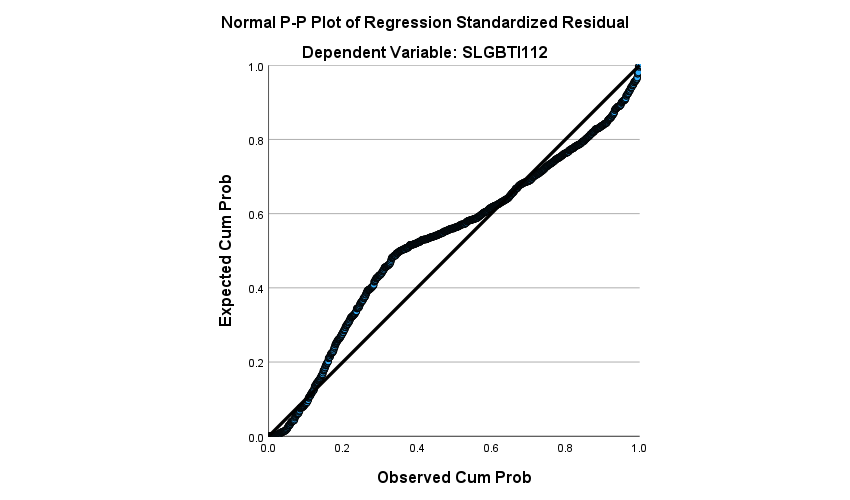


| **ANOVA^a^** | | | | | | |
| --- | --- | --- | --- | --- | --- | --- |
| Model | | Sum of Squares | df | Mean Square | F | Sig. |
| 1 | Regression | 11.393 | 2 | 5.696 | 3.744 | .024^b^ |
|  | Residual | 1518.215 | 998 | 1.521 |  |  |
|  | Total | 1529.608 | 1000 |  |  |  |
| 2 | Regression | 460.171 | 5 | 92.034 | 85.628 | <.001^c^ |
|  | Residual | 1069.436 | 995 | 1.075 |  |  |
|  | Total | 1529.608 | 1000 |  |  |  |
| a. Dependent Variable: SLGBTI112 | | | | | | |
| b. Predictors: (Constant), Sex_Aut=Female, Age | | | | | | |
| c. Predictors: (Constant), Sex_Aut=Female, Age, CVO, PHI_12, RBO | | | | | | |


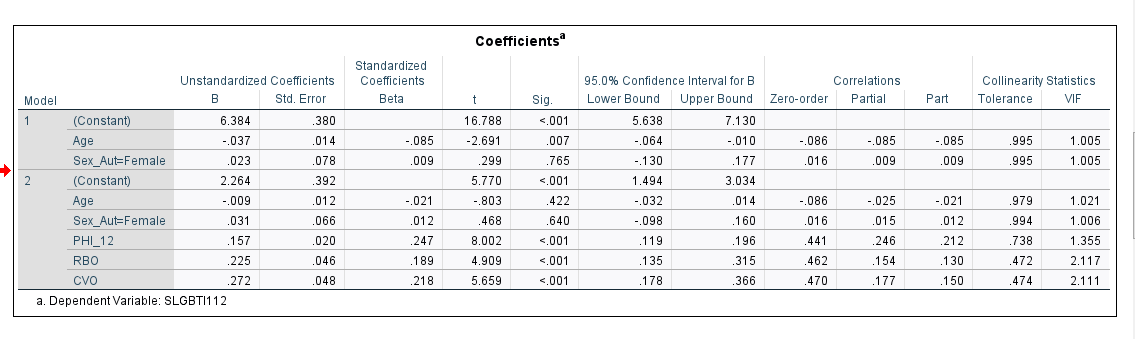

Supplement: S3 File — (DOCX) [file pone.0287726.s003.docx]
