# Supplementary material for: Lesbian, gay, bisexual, transgender, intersex, and its legalisation in Africa: Insights from tertiary-level students in Ghana
Source: PLoS One. 2023 Jul 7;18(7):e0287726. doi: 10.1371/journal.pone.0287726 (PMC10328334; doi:10.1371/journal.pone.0287726)
Supplement: S1 Appendix — (DOCX) [file pone.0287726.s004.docx]

**LATENT VARIABLES ASSESSED IN THE STUDY**

**Support for the Passage of Anti-LGBTI**

1. In general, to what extent do you agree or disagree with the passage of laws against LGBTI individuals (anti- LGBTI laws) in the country? (SLGBTI1)
2. Do you think there is a need to pass such kinds of laws (anti-LGBT laws) in this modern era? (SLGBTI2)
3. To what extent do you agree or disagree that there should be laws to prevent mature individuals (i.e., over age 18) from choosing the gender they prefer to be identified with? (SLGBTI3)
4. Mature individuals who engage in same-sex sexual activity should be legally punished? (SLGBTI4)
5. To what extent do you agree or disagree that there should be laws that prevent mature same-sex individuals from marrying? (SLGBTI5)
6. To you, the passage of anti-LGBTI laws (laws against LGBTI) in the country has been delayed too much? (SLGBTI6)

**Religious Beliefs**

1. Is your religious denomination likely to warmly accept LGBTI people (RB1)
2. To what extent is your religious denomination likely to accept LGBTI-related activities (e.g., same-sex marriage)? (RB2)
3. How likely are you to find identified LGBTI persons in your religious denomination? (RB3)
4. To what extent is identified LGBTI individual likely to become a leader in your denomination. (RB4)

**Cultural Values**

1. LGBTI people are likely to reveal themselves freely in your community. (CV1)
2. An identified LGBTI individual is likely to be accepted to participate in your community’s cultural activities. (CV2)
3. To what extent do you think LGBTI-related activities (e.g., same-sex marriage) are likely to be welcomed in your community? (CV3)
4. To you, how likely can an LGBTI identified person be selected to mount a leadership role in your community? (CV4)

**Perceived health Implications of LGBTI and related activities**

There should be passage of laws against LGBTI because...

1. Sexual intercourse between homosexuals is more risky than sexual intercourse between the opposite sexes in contracting sexual related diseases. (PHI1)
2. Homosexuals are mostly less healthy than heterosexuals. (Healthy used here means having good physical and mental condition) (PHI2)
3. All LGBTI individuals are suffering from one disease or the other (the diseases could either physical, mental or both). (PHI3)
4. LGBTI individuals have higher tendency of sharing diseases among the populace. (PHI4)
